# From benefactor to outsider: conditional personhood and the withdrawal of recognition after sudden wealth in an unequal, collectivist context

**DOI:** 10.3389/fsoc.2026.1853012

**Published:** 2026-08-25

**Authors:** Lusanda Sekaja, Tshegofatso Mabitsela-Siwela, Esmarié Petro van der Merwe

**Affiliations:** Department of Industrial Psychology and People Management, University of Johannesburg, Johannesburg, South Africa

**Keywords:** African communitarianism, belonging, identity, relational personhood, social positioning, social recognition, sudden wealth

## Abstract

**Introduction:**

Scholarship on sudden wealth has typically focused on financial mismanagement and psychological adjustment, with little attention to its impact on identity and social recognition in unequal collectivist contexts.

**Methods:**

Guided by African communitarianism and theories of recognition and social positioning, we draw on a qualitative analysis of narratives from the South African documentary series I Blew It to examine how sudden wealth reshapes personhood within community life and how recipients are morally evaluated and repositioned, both while resources are plentiful and once they diminish.

**Results:**

The findings revealed that sudden wealth attracted community gaze, increased recipients’ social recognition, and placed expectations on individuals to share their resources to secure the community’s approval. As the funds are depleted, recognition is withdrawn, and the recipients are mocked or avoided. This leads to shame and a disrupted sense of self.

**Discussion:**

These findings suggest, at least in unequal collectivist societies, that personhood is not a given and depends on the continued distribution of resources. By linking identity theory to African communitarianism, this study demonstrates that while personhood can be affirming, it can also be extractive. When sudden wealth is introduced into the social dynamic, it reshapes recipients’ identities. This study makes three contributions: extending theories of cultural processes, extending work on recognition claims, and challenging idealized accounts of African communitarianism. Although grounded in South Africa, the findings offer theoretical insights that may be transferable to other unequal, collectivist societies, because the mechanisms we identify, namely conditional personhood, withdrawal of recognition, and communal gaze, are likely to emerge wherever material scarcity and strong communal obligations coexist.

## Introduction

Sudden wealth changes individuals’ financial circumstances, alters how they are perceived within their communities, and reshapes their sense of belonging. In South Africa, these changes are vividly portrayed in the long-running reality documentary *I Blew It*, which follows people who unexpectedly receive large sums of money and later face the emotional and social consequences of their financial choices (Molokoane, 2024; Mpofu et al., 2023; Ngoepe-Ntsoane, 2022). The show illustrates how recipients’ actions shift from restraint to extravagance and how their positions within social networks evolve once they are perceived as wealthy. In a society marked by high unemployment and deep inequality, such stories attract public interest because they speak to both aspiration and caution (Faluyi and Olutola, 2024; McKeever, 2024). The narratives of participants of the show construct sudden wealth as an event that alters the recipient’s social position in the community, and by extension, their belonging and social recognition.

Although international studies have examined how sudden wealth affects wellbeing, they have tended to focus on psychological outcomes in middle- and upper-income contexts (e.g., Chai, 2021; Hedenus, 2011; Larsson, 2011; Raschke, 2019). Furthermore, previous research has identified patterns of overspending and financial mismanagement but has seldom explored the accompanying shifts in social identity and belonging that arise when someone moves from dependency to temporary affluence (Ferraro et al., 2013; Sherman et al., 2020; Van Wyk, 2012). South Africa is marked by high inequality (Francis and Webster, 2019), and Black South Africans tend to hold collectivist values (Eaton and Louw, 2000) that guide their behavior. These patterns interact to create unique social dynamics.

In African contexts, identity and recognition cannot be constructed without considering who holds power and material resources (Mama, 2001). In such unequal contexts, sudden wealth creates moral expectations for economic support. Behavioral changes, social pressure, and identity shifts are intertwined with how people are viewed and with the moral judgment from the community (Ferraro et al., 2013; Nosike and Ojobor, 2024; Ventura, 2025). While attaining sudden wealth can be empowering, as it offers freedom from the burdens of financial constraints, it also has the potential to destabilize relationships and social standings by highlighting conditional forms of belonging that can be withdrawn at any time.

Existing research on sudden wealth has focused on financial mismanagement and psychological adjustment, typically in individualistic, high-income contexts. Sherman et al. (2020) developed a dynamic model of happiness and documented lottery winners’ patterns of overspending. Apouey and Clark (2015) and Lindqvist et al. (2020) assessed the mental and physical health outcomes associated with lottery prizes and found mixed effects on life satisfaction. Ferraro et al. (2013) examined conspicuous brand use as a form of individual signaling behavior, while Larsson (2011) explored how lottery winners construct identities through consumption. Hedenus (2011) and Raschke (2019) considered psychological wellbeing and the subjective experience of sudden wealth. However, their samples were drawn from Sweden and Germany, respectively, which are contexts characterized by individualism and low inequality relative to South Africa.

Although those studies have generated important insights, they pay little attention to the social processes by which people gain or lose recognition within their communities: whether sudden wealth may reshape identity through social recognition, or how belonging may be granted conditionally and later withdrawn by the community in unequal, collectivist settings. In contexts where material resources are scarce and communal obligations are strong, recognition may become tied to the continued distribution of resources. Thus, personhood becomes fragile and revocable.

We therefore asked: How do people who have experienced sudden wealth and its loss narrate changes in their identity, social recognition, and belonging? Drawing on selected episodes from the documentary series *I Blew It*, we analyzed how recipients of sudden wealth described how their communities saw and treated them and how they made sense of their moral worth before, during, and after their financial decline.

To address gaps identified in previous studies, we draw on two bodies of literature that have not yet been applied to sudden wealth research. First, the *cultural sociology of inequality* shows that recognition, that is, being seen as a valued, worthy member of society, is shaped by others’ judgments of an individual (Lamont et al., 2014). However, this literature has focused largely on how recognition is contested, not on what happens when it is withdrawn. Second, *African communitarian philosophy* emphasizes that personhood is relational and earned through moral participation in community life (Molefe, 2018; Oyowe and Yurkivska, 2014). However, this framework has not been tested in contexts where material resources change rapidly, which may expose the conditional nature of belonging. Our study attempts to bridge these bodies of literature by examining sudden wealth as a period when recognition is granted and then withdrawn. This makes the conditional and extractive nature of personhood in unequal collectivist contexts more visible.

### The social basis of identity: recognition, belonging, and moral worth

Recent studies in the cultural sociology of inequality offer a useful lens for understanding these processes. Recent sociological research has moved beyond viewing inequality solely in terms of material resources and increasingly examines the cultural processes through which worth, dignity, and social inclusion are allocated (Lamont et al., 2014). From this perspective, inequality is not only about who has resources but also about who is recognized as respectable and deserving in society. Recognition is the social validation of identity by others and plays an important role in shaping belonging and social standing. Lamont et al. (2014) argued that inequality is produced through processes of identification and evaluation. People are judged and assigned social value using shared cultural beliefs about what is desirable, respectable, and morally appropriate. These processes shape who is included, who is excluded, and whose contributions are valued. In their comparative study of responses to stigmatization, Lamont et al. (2014) showed that recognition is actively negotiated: Individuals draw on cultural repertoires and boundary work to respond to devaluation and claim worth.

More recently, Kahana and Schmid (2026) demonstrated that people actively negotiate recognition when others challenge their moral worth. They tend to make recognition claims about their worth and, when challenged, they may negate claims to try and discredit their critics. Kahana and Schmid’s research demonstrates that recognition is not just granted or withheld but is also defended and negotiated. Building on this scholarship, we examine what happens when recognition is withdrawn following a sudden decline in wealth.

Other scholars have examined how elites legitimize their position through cultural work. Kantola and Kuusela (2024) showed that wealthy elites craft shared establishment fantasies or collective narratives that justify their political interests and moral standing. Similarly, Kuusela (2022) introduces the concept of hyperopia of wealth to describe the discursive blindness that top earners have toward the structural conditions of inequality, ignoring the role of wealth accumulation in creating disparities. Research on the wealthy in other contexts has also shown that affluence brings social pressures, expectations of generosity, and shifts in identity (Kuusela, 2022; Sherman, 2017). These studies show that cultural processes of recognition are central to the production and reproduction of inequality.

Our study builds on this body of research by examining a population that has received less attention in the literature on recognition and inequality: individuals in a highly unequal, collectivist context who experience sudden wealth. While Lamont et al. (2014) focused on how marginalized groups respond to stigmatization, we examine how sudden wealth recipients experience shifts in recognition, first elevated and then withdrawn. This allows us to extend theories of recognition beyond the study of stigma and marginalization to consider how recognition operates in contexts of rapid material change. Sudden wealth provides a useful context for examining these processes because it rapidly alters an individual’s access to resources and social position. Community members may begin to view the recipient differently and expect them to behave in ways that align with local norms of generosity and reciprocity. In highly unequal settings, recognition may become closely tied to the sharing of resources and the fulfillment of social obligations.

### African communitarianism: from relational personhood to conditional belonging

African communitarianism serves as the theoretical lens through which we understand personhood in this study. The socioeconomic realities prevalent in South Africa constitute the context that shapes how these normative ideals are carried out. It is important to distinguish among three levels of analysis. First, African communitarianism is a philosophical tradition that emphasizes the relational nature of personhood: Identity is constituted through community, and moral worth is earned through participation in social life (Molefe, 2018; Oyowe and Yurkivska, 2014; Oyowe, 2015). This is a normative ideal, that is, a vision of how human beings ought to relate to one another. Second, the socioeconomic realities of contemporary South Africa, characterized by high unemployment and material scarcity, shape how these ideals are practiced (Francis and Webster, 2019). In contexts of scarcity, communal expectations may become more demanding, and the ability to share resources may become a more visible measure of moral worth. Third, our study examines specific narratives of sudden wealth recipients as represented in the documentary series *I Blew It*. These narratives reflect their socioeconomic realities and show how communal expectations operate in one particular context where inequality is high and resources are scarce.

Within African communitarian thought, identity is not a fixed personal attribute but a socially conferred status continually affirmed or withdrawn through interactions with others. In this way, African communitarianism may be understood as a culturally specific system of recognition by which personhood is granted and continually evaluated by the community in which the person lives. Accordingly, an individual shapes and actualizes their identity through relationships and engagements with the community. Unlike individualistic ways of being, African communitarianism emphasizes that connectedness with others gives people’s lives meaning and dignity (Oyowe and Yurkivska, 2014). Scholarship agrees that African communitarianism introduces personhood, a process of becoming earned and maintained through relationships with others (Bayuo, 2025). This means that personhood must be demonstrated through actions the community recognizes as morally good.

Personhood is relational. It is achieved, recognized, and maintained through behavior that is socially valued and can thus become a tool of discipline, not just of belonging (Bayuo, 2025; Molefe, 2018; Oyowe and Yurkivska, 2014). Individuals in the community must grow into their personhood through responsible participation in social life (Molefe, 2018). While the rights of the individual are important, they are secondary to social responsibility, and only by fulfilling one’s duties to society can one’s personhood be recognized. Conversely, moral failure and neglect of social responsibilities can lead to diminished personhood. In these cases, exclusion is not accidental but a tool to enforce and even demand desirable norms and conformity in the community. By excluding a person, the community signals a withdrawal of recognition, thereby reducing that individual’s moral standing in the communal social hierarchy. African communitarianism is therefore criticized for depicting communities as morally unified, when in reality, contestation and inequality exist (Oyowe, 2015). When a society is unequal, a communal expectation to share resources may be heightened, and in so doing, makes personhood dependent on one’s ability to fulfill these expectations. This is particularly true when considering that it cannot explain conflict, exclusion, and failure in a community.

African communitarianism may present personhood as something an individual earns by acting in accordance with the needs and desires of the community; however, Mama (2001) disputes romanticized depictions of community as a supportive and unified entity. Identity discourse tends to mask internal power struggles and inequalities, and what may be perceived as shared moral expectations may reproduce coercion and exclusion. This points to community as a site marked by scarcity and power, especially in the context of inequality. While communitarian personhood is affirming and humanizing, it can also be disciplinary and conditional on meeting communal expectations.

### Wealth as a moral obligation

This study analyzes how people narrate their behavior after gaining sudden wealth, and how these narratives construct identity, recognition, and belonging. Various factors account for changes in their spending behavior, including external (family, community) pressure and mismanagement of wealth (Sherman et al., 2020).

African societies place significant value on giving or generosity toward family members or the community (Oppel, 2023; Ventura, 2025). Individuals feel an obligation to spend in service of others, especially when there are expectations of family provision and social reciprocity, which position generosity as a moral obligation rather than a voluntary act (Mnisi, 2015; Oppel, 2023; Sharif and Khanekharab, 2017). In this context, the attainment of wealth is both a personal gain and a communal resource. A recent study by Talhelm et al. (in press) points to how relationships are maintained through fulfilling responsibilities to family and other important people in one’s life. Sudden wealth therefore enters a social world in which recipients may be expected to meet obligations that are understood as moral duties rather than discretionary acts of generosity.

Belonging has been positively connected to giving across various settings. For instance, Drezner and Pizmony-Levy (2021) found that a sense of belonging among graduate students predicted alumni giving to their alma mater, while McClure and Ryder (2018) demonstrated that college students spend money strategically to influence social relationships. Jiang (2023) showed that charitable giving among immigrant populations was a way for them to attain belonging. Broadly, belonging is understood as affective and symbolic (Çolak et al., 2025; Imboden, 2024).

In this context, identity is negotiated relationally rather than individually, and one’s acceptance depends on how one conforms to the communal expectations. Sudden wealth therefore reorganizes the expectations placed on the individual and their social position, effectively changing one’s identity. Therefore, cultural expectations may be important in understanding one’s identity within the community after sudden wealth attainment.

### Behavioral responses and conspicuous consumption as social signaling

Instead of or in addition to spending on family members or the community, several individuals adopt lavish lifestyles to symbolize a change in social class or perception of identity. These public displays are attempts to gain communal respect; however, they inadvertently expose individuals to criticism and judgment.

Introduced by Veblen (1992), the concept of conspicuous spending refers to consumers’ purchasing goods or procuring services (that they may not be able to afford) as a public declaration of status. Cultural and social factors influence conspicuous spending. The practice is often motivated by social competition (Wang and Griskevicius, 2014), and its intended goal therefore is for individuals to show off their success and secure their position in the community’s social structure. Individuals may feel obliged to display an affluent lifestyle as a means of sustaining identity legitimacy (Falk and Graeber, 2020). These behaviors are identity performances through which individuals seek recognition and belonging (Han et al., 2010). Conspicuous spending is both a display of status and the ongoing pursuit of approval and the maintenance of a good reputation under the watchful eye of the community.

Individuals belonging to lower-income communities may display their status or material wealth in their extended family or community because of pressure to symbolize “success” (Dondolo and Madinga, 2017; Nelissen and Meijers, 2011). In this way, the way people spend their money is a social act aimed at others rather than themselves. Individuals may conform to or evolve into accepting these spending behaviors because of status norms and expectations from their social environment (Frank, 2007). This process shows how identity is negotiated publicly through public displays of excess.

Individuals from lower-income communities in South Africa tend to mimic an affluent lifestyle, such as owning expensive houses or vehicles and wearing designer clothing, in an attempt to gain social acceptance in their communities (Dondolo and Madinga, 2017). Conspicuous consumption becomes a means of achieving recognition, a direct reflection of Veblen’s early notion of expenditure as a display of status and prestige (Baker and Widmaier, 2014). In the context of this study, conspicuous spending is therefore interpreted primarily as social signaling rather than irrational financial choices. People’s behaviors after receiving sudden wealth should be viewed as strategies shaped by their social world in an attempt to secure acceptance, respect, and moral standing within the community.

### Psychological effects and identity negotiation

The behavioral patterns associated with sudden wealth, including impulsive buying, substance use, and social spending, reflect changes in individuals’ psychological and emotional functioning after acquiring it (Ferraro et al., 2013; Sherman et al., 2020). The effects of a sudden windfall may also extend beyond spending behavior, reshaping other aspects of identity. In a related study of the same documentary series, Van der Merwe et al. (2026) found that recipients often disengaged from work after acquiring sudden wealth and increasingly defined themselves through visibility, social status, and obligations to others. Rather than deriving meaning from occupational roles, the participants became recognized as benefactors and influential community members. This suggests that sudden wealth may reorganize multiple identities simultaneously, with community recognition becoming more salient than work-based identities. This shift toward community-based recognition may explain why belonging becomes closely tied to giving.

Individuals who acquire sudden wealth often experience fear or psychological and emotional distress because they feel that they came into money solely based on “luck” (Jaffe and Grubman, 2007; Sahi and Dutta, 2015), especially in low-income contexts (Raschke, 2019). However, this is not a universal finding, as other scholarship has determined either that windfalls can increase life satisfaction and mental health slightly (Apouey and Clark, 2015; Lindqvist et al., 2020) or that they have no impact on long-term health (Östling et al., 2020). Feeling that their success is unearned drives individuals to prove their success through conspicuous means that the community can recognize. From an identity perspective, these behaviors can be read as attempts to sustain visibility and recognition under conditions of heightened communal scrutiny.

Once those resources decline, however, the social roles associated with generosity, provision, and influence collapse, often resulting in emotional deterioration, anxiety, and depression (Apouey and Clark, 2015; Sherman et al., 2020). This collapse is about more than simply financial loss. It also represents a loss in both social standing and a stable sense of self. Individuals often express feelings of frustration or resentment when they lose wealth (Lindqvist et al., 2020; Pronello and Gaborieau, 2018). The financial decline often initiates changes in an individual’s lifestyle and wellbeing. The return to dependency or unemployment thus represents a loss in social standing and personhood within the community.

The consolidation of the triad of the concepts of identity, recognition, and African communitarianism shows personhood to be communally granted and regulated. Sudden wealth may elevate identity by momentarily increasing recognition and expectations of sharing resources, which, if not fulfilled, may prevent belonging from being granted. Extant research attaches financial decline mainly to mismanagement and rarely ties it to the withdrawal of recognition and repositioning in communal hierarchies. We sought to fill this gap by exploring how financial windfalls and loss thereof reshape identity and recognition in South African communities.

## Materials and methods

### Method

Through an analysis of the television series *I Blew It*, we sought to understand how participants and other members of their social world make sense of recipients’ identities and community belonging, and how they confer recognition on recipients as recipients navigate the various stages of their wealth. We therefore adopted a social constructivist–interpretivist philosophy, which assumes that meaning is made through human interaction and one’s social and cultural contexts (Ugwu et al., 2021). In this study, identity is conceived as formed through relationships and social interactions. We analyzed how individuals are positioned within the family and community and closely examined the sense of obligation the recipient may have toward them and how they may be acknowledged, judged, and disconnected from these social groups. All the episodes of *I Blew It* recount stories of individuals who received windfalls in the millions of rands through the Road Accident Fund, inheritance, or sports or acting deals. Although the data were not produced for the study and may have undergone intervention for television production purposes, identity, belonging, and recognition are nevertheless reflected and negotiated in the narratives of the recipients, their family, friends, and colleagues. Importantly, the stories (the source of sudden wealth and the basic details of spending and decline) have been verified by the production team.

At the time of data collection, 78 episodes of *I Blew It* were available across six seasons. All episodes were screened for the study’s inclusion criteria. Episodes were included if they met the following criteria:

- Featured an adult recipient who had experienced sudden wealth,
- Contained narrative accounts of life before, during, and after wealth acquisition, included discussion of relationships with family members, friends, or the wider community, and
- Provided evidence of changes in identity, social recognition, belonging, and/or communal expectations following the windfall.

This screening process identified 10 episodes suitable for in-depth analysis. Rather than including all episodes, we purposively selected these information-rich cases for their clearest and most comprehensive accounts of identity transformation, community recognition, belonging, and the consequences of wealth decline. The selected episodes also included perspectives from recipients as well as family members, friends, colleagues, and community members, allowing for a richer examination of how recognition and personhood were negotiated. Episodes that did not provide this were excluded. Variation in the source of wealth and social circumstances was considered during selection to maximize the breadth of experiences represented in the final sample. Non-conforming episodes were not considered for analysis. Following Sekaja and Kgosiemang (2026), we regard participation as a relational process. We therefore treated the documentary narratives not as simple truth, but as accounts shaped by participants’ desire for their stories to matter to their community. In this study, we treat the televised narratives from *I Blew It* as sites where community recognition and personhood are performed and negotiated under sudden wealth. Table 1 provides an overview of the primary participants, detailing both demographic information and a categorized summary of their sudden wealth arc from source to decline.

**Table 1:** Participant overview.

Participant	Age at interview	Primary recognition mechanism	Communal expectation	Boundary attempt	Communal reaction post decline	Family repositioning and identity consequence	Additional interviewees
P1	42	Television visibility; celebrity status; community admiration; financial provision; conspicuous spending	Provide financially for family; sustain generosity; maintain visible success; host and fund lifestyle activities	No explicit boundary attempt stated; continued financial support and spending until decline	Laughed at; gossip; reduced calls; celebrities stopped visiting; public ridicule; stigma as addict	Family treated him like a child; locked him outside house; did not trust him with money; brother repaid debts; partner left with children; loss of respect; identity marked by shame and regret	Sister, brother, manager, friend
P2	26	Conspicuous spending (cars, luxury clothing, club money showers); generosity; visible cash circulation; music fame identity	Provide for parents; build family house; assist with funeral costs; donate food; maintain social generosity and status display	Initial structured investment (R2 million [US$118,000] fixed-term; staged withdrawals), but later withdrew investment to fund spending and business purchases	Labelled reckless; perceived as gangster; social fascination; later disbelief when money depleted; reduced admiration	Conflict with mother over house funds; father unaware of depletion; eventual financial regret; identity reframed as lesson learner	Father, friend 1, friend 2
P3	24	Luxury clothing; VIP transport; five-star hotels; party hosting; visible cash spending; music video exposure	Support mother; purchase stand and build house; provide for siblings; maintain generous host role	Initial grocery restraint; attempted stand purchase; later impulsive withdrawals and theft of bank card	Gossip; ridicule; loss of “landlord” status; laughter; public commentary on waste	Loss of family stand; maternal disappointment; remorse; emotional distress; identity marked by regret	Mother, brother, friend 1, friend 2
P4	33	International athletic success; Olympic medal; luxury cars; VIP club dominance; cash distribution; visible drug-fueled excess	Support family financially; build/extend family home; inspire township youth; act as community icon	No sustained boundary; continued drug use despite prior suspension; ignored family warnings; financial manager alerts disregarded	Public gossip; loss of icon status; suspension; stigma; community disappointment; rumors about causing mother’s death	Mother’s illness and death; father’s decline; remorse; rehab attempts; living in abandoned building; loss of assets; desire for comeback	Sister, uncle
P5	35	Conspicuous spending (cars, rims, sound system, alcohol, champagne); cash gifting; club dominance; proposal spectacle; visible generosity	Provide for parents and siblings; build house; sustain daily generosity; act as township success symbol; constant hosting and alcohol provision	Initial business attempt (mobile kitchen); small fixed withdrawals; but ignored business advice; withdrew investment; consumed business stock for leisure	Gossip; nickname reduced to mockery; loss of “worshipped” status; social avoidance; fear of walking in streets due to ridicule	Relationship breakdowns; eviction from flat; reliance on new partners; family disappointment; emotional distress; eventual self-reflection and modest reintegration	Brother, friend 1, friend 2
P6	33	Multiple Audi A3 vehicles; branded clothing; expensive liquor; three-day house parties; alcohol; casino gambling; visible cash carrying; drug use	Maintain affluent lifestyle; host social gatherings; uphold family status; visible consumption aligned with prior well-off upbringing	None sustained; continued gambling and daily drug spending; withdrew final R200,000; sequential sale of assets	Mockery (“laughing stocks”; “hobos”); community gossip; social distancing; visible deterioration (torn clothes); shame; some community members happy about decline	Sold family home; relatives gave up; younger brother entered rehabilitation; older brother still using; unhoused (approx. 6 months); regret over selling family legacy; partial family support from friends	Brother, friend 1, friend 2
P7	Not stated	National soccer fame; luxury cars, house for mother; VIP nightlife dominance; alcohol spending; visible financial generosity	Provide for extended family; cover funeral costs; buy groceries (R5,000+); maintain township friendships; sustain high-status social presence	No sustained boundary; continued alcohol abuse; debt accumulation; relied on partner to manage finances; investments cashed out to cover bills	Mockery; labelled “laughing stock”; public gossip; loss of respect; eviction; auction of houses; reliance on small handouts	Family displaced; furniture repossessed; mother and relatives distressed; loss of contact with child; shame; withdrawal from public soccer spaces	Mother, brother, aunt, friend
P8	Not stated	Music fame; media visibility; stadium performances; political bookings; luxury purchases	Support family; maintain moral/religious image; sustain generosity and status	No sustained boundary; escalated alcohol and cocaine use; missed gigs	Public scandal; dismissal by production company; media shame; unhoused; labelled failure	Minimal financial support to mother; children placed in foster care; later apology and reconciliation; identity reframed through rehab	Mother, friend 1, friend 2, rehab employee
P9	29	House purchase; cars; business ownership; jewelry; visible consumption; generosity to siblings	Provide for siblings; support parents; buy house; host thanksgiving; act as family stabilizer	Initial R100,000 fixed account; later early withdrawal with R30,000 penalty to cover expenses	Friends distanced; public criticism (“foolish with money”); loss of status; reduced social contact	Sold house at loss; marital strain; rental eviction; family relocation; depression; employment under supervision	Wife, Brother, Mother
P10	38	Entrepreneurial success; award recognition; visible relocation to Sandton (upmarket suburb in Gauteng); conspicuous spending	Grow business; uplift family; sustain township pride; act as role model	Circumvented spending limits; concealed parallel business; ignored oversight	Mockery; labelled “hobo”; gossip; legal complaints; social withdrawal	Neglected family provision; sold possessions; weight loss; rehab admission; later cautious reintegration	Friend 1, friend 2, sister, aunt

The participants were Black South African men who experienced sudden wealth from sources such as Road Accident Fund payouts, professional sports, entertainment careers, insurance benefits, and business ventures. Across cases, recognition was typically linked to visible success, generosity, and financial support for others. Participants were expected to support family members, maintain an affluent lifestyle, and contribute to their communities. Following financial decline, many faced social distancing, ridicule, loss of status, and strained family relationships, making the sample well suited for examining how identity, belonging, and recognition were negotiated through sudden wealth and its loss.

### Data analysis

We analyzed data from the show using reflexive thematic analysis [following the recommendations of Braun and Clarke (2019), Braun et al. (2023)], focusing on how participants negotiated their identities through social interaction. We were particularly interested in how participants were positioned by others in their lives and how they framed changes in obligation and value to the community over time. Initial coding captured actions undertaken by the participants when they received their windfall (e.g., spending and visibility). These initial codes were then closely examined for what they revealed about shifts in social positioning. In this way, spending was interpreted less as a financial behavior and more as campaigning for social recognition. Similarly, when the funds were depleted, it was not conceptualized as a failure in financial management. Instead, it was viewed more in relation to the ways the recipients then withdrew from society. Codes were then grouped to reflect patterns in identity processes, including elevation, expectations, and withdrawal. In the later stages of the analysis, African communitarian ideas of relational personhood were used as the interpretive lens that helped us make sense of how identity was granted and withdrawn through communal expectations. We identified and refined themes through researcher consensus and named the themes based not just on common patterns but also on moments that disagreed with dominant interpretations.

### Researcher reflexivity and positionality

Given the interpretive nature of this study, we acknowledge that our positions and assumptions shaped the research process. The research team consisted of two Black South African women academics and one White South African postgraduate student, who collected the data. Our varied positions provided both insider and outsider perspectives on the narratives analyzed. The Black academics brought contextual understanding of South Africa’s inequality and collectivist norms, while the White student, though positioned differently, engaged reflexively with her assumptions about wealth and community throughout the data collection and analysis. None of us has personal experience of sudden wealth, and we recognize that this may have influenced our interpretations of the participants’ decisions and behaviors.

To manage the risk of imposing our views onto the data, we engaged in ongoing reflexive dialogue throughout the analysis. We regularly questioned our assumptions and considered alternative interpretations of the data to ensure that the themes remained grounded in the participants’ accounts. Of course, as research instruments ourselves, our subjectivity can never be truly removed from our interpretation. Multiple team discussions and investigator triangulation further helped to balance our individual perspectives and reduce the influence of any single interpretive bias. We also recognize that our use of documentary narratives and our consumption of the show *I Blew It* prior to taking part in this study required us to be mindful of how production choices and editorial framing shaped the stories we analyzed. Throughout the analysis, we remained aware that we were interpreting mediated accounts rather than direct records of experience, and we approached the narratives with sensitivity to the participants’ dignity and cultural context.

## Results

In this section, we present four themes that address our research question: how people who experienced sudden wealth and its loss narrated changes in their identity, social recognition, and belonging. The themes trace a trajectory from elevation to decline. The first captures how participants described being recognized and celebrated by their communities. The second theme comprises the participants’ narrations of the obligations that came with this recognition. Under the third theme, we present participants’ accounts of how they were regulated through the communal gaze. The fourth theme tells the stories of exploitation, shame, and the withdrawal of recognition.

### Theme 1: communal relationships shape and validate identity

After attaining sudden wealth, the participants began to construct their identities around a robust social life of friends and neighbors who put themselves in proximity to the recipient, even when there was no prior relationship between them. The following participant quotes describe the sudden social prominence many recipients experienced:

*“He was a celebrity, so everyone wanted to take pictures with him. The whole community loved him.”* (Sister of P1).

*“Today, you are nobody; tomorrow, you are something big. You cannot walk freely in malls anymore. Moreover, he caused havoc because those characters became like gods.*

*Everywhere they went, I have never seen something like that.”* (Manager of P1).

People would shower the recipient of sudden wealth with admiration, which validated their high standing in the social hierarchy. In line with African communitarianism, through this validation, the participant was granted personhood through social recognition. They also placed expectations on the recipients by making them providers of material goods and good times. Through their sudden wealth, the recipients became the subject of communal gaze and were watched and admired fanatically. Praise from the community demonstrated that their elevated social identity was by no means self-bestowed but instead granted to them by the community. This shows the relational nature of personhood.

*“Everyone back home called him the landlord. Listen to that: the landlord. As the landlord, he owns every place he goes. When he gets to a party, he takes charge of the whole party.”* (Friend 1 of P10).

*“When we went to the club, everybody was screaming his name.”* (Friend 2 of P3)

*“People worshipped him.”* (Brother of P3).

Such musings from friends and relatives serve to show how identity is elevated through communal naming and symbolic status. This suggests that both identity and personhood are produced through shared social meaning and not necessarily through achievement or some other merit of the individual. Even the members of the community who might not previously have been on good terms with the recipient would change their attitudes toward them and view them in a more favorable light.

*“The neighbors who did not like you now like you.”* (Manager of P1).

Such a shift demonstrates that belonging and personhood are not guaranteed; they are conditional upon how the community perceives one’s contribution and value to the collective. The recipients’ sudden wealth repositioned them as high-value individuals, reinforcing the notion that identity is granted relationally: communities re-categorize individuals who acquire wealth by assigning them a new social identity. The recipient was no longer seen as an ordinary community member but as a “celebrity” or “landlord,” which are statuses that carry admiration and expectation. This re-categorization was not neutral because it increased the recipient’s social standing while simultaneously creating new obligations. The community’s praise and attention indicated that the recipient’s value was now tied to their ability to provide, which set the stage for future expectations.

### Theme 2: obligations to the community

This theme shows that wealth was considered not as something to be possessed by a single individual but as something that the recipients had a duty to share with others around them. In this case, wealth was a relational asset that granted the participants a sense of belonging and personhood. The people who surrounded the recipient expected that their windfall would be redistributed to them in some form or another. Generosity was expected from the recipients, and they were seen as having a moral duty to share their good fortune. The extent of wealth shared was a measure of social belonging and moral standing: The act of giving solidified relationships, and refusing to do so violated a relational code of ethics. In the opinion of community members, wealth is something that belongs to the community and should be shared with others. If an individual fulfilled this expectation, they were granted personhood. It would appear that both the giver (of money, goods, or experiences) and the recipients understand an unwritten rule that there was no obligation for the beneficiaries to pay it back. This lack of obligation to reciprocate strongly suggests that sharing wealth with one’s community is considered a moral duty and not a transaction.

The participants acted on this communal expectation by giving money and purchasing goods and experiences for family, friends, and community members. Acts of generosity serve to affirm one’s identity. This allowed recipients to show that they were valued, ethical members of society. An example of giving to the community came in the form of one recipient’s donating food packs to members of the community whom he knew from the time he had a spaza shop (a small, informal, often home-based convenience store found in South African townships that sells everyday essentials to the local community) and who he knew were unable to sustain themselves. He also showed generosity to unhoused community members by paying someone to cook for them. The recipient recounted the following:

*“You could tell when someone came to ask for credit that this person is really hungry. That used to hit me emotionally, even with the kids; you could tell that their home situation was tough. So that is when I decided to gather my friends and donate food to the people. I withdrew R100,000 [about $6,000]. We bought food and vegetables and donated food packs. We took the food to different homes. We cooked meals to feed the homeless, and I had hired people to do all that work.*

*I paid them about R20,000 [about $1,200]. There were four of them”* (P2).

Generosity was a way for recipients to respond to the needs of the community, and by doing so, retain recognition and personhood. Family members were often the greatest beneficiaries of the windfall of the recipients, who would pay for their necessities and means of upskilling themselves. It may therefore be said that family was the site where communitarian obligations were most felt and obliged. Again, there was no expectation from either party that the funds be returned. While this may be seen as an act of doing good, it can also be construed as an expression of kinship obligation.

*“I gave each of my brothers R30,000 [about $18,000]. Whatever they needed, I gave it to them. I bought them clothes and shoes. I took them for the boiler-making certificate.”* (P9).

His brother confirmed receiving this money:

*“Yes, at first, he gave me some cash. He gave me about R30,000. However, then after that I wanted to borrow some cash because I was down. So, I think I took about R15,000 [$9,000] from him. I think it ended up like R40,000 to R45,000 [$2,400 to $2,700]. I never repaid him.”* (Brother of P9).

From an African communitarian perspective, this theme shows that fulfilling family and community obligations monetarily was a means of affirming personhood, and conversely, failing to do so was a risk to social standing and belonging. This reveals how wealth becomes converted into moral obligation. The community evaluated the recipient’s worth based on their willingness to share resources. Giving seemed to be a measure of whether the recipient was living up to their new social standing. If a recipient refused to give or gave reluctantly, they risked being judged as selfish and failing their moral responsibilities to the community. Thus, generosity became the primary standard by which the recipient’s personhood was assessed.

### Theme 3: communal gaze regulates behavior

The windfall placed recipients under constant community gaze, which functioned as a way for the recipient’s behavior to be judged and their identity regulated. When a recipient acted in accordance with what was expected of them and what was good for the community, these actions garnered praise, and the individual was granted personhood. However, when one abused and depleted their fortunes, the community cast them in shame and thus withdrew their personhood. This again demonstrates the conditional nature of personhood, which is socially, not automatically, granted and fulfilled. The recipients’ recognition depended on their ability to provide material benefit, and once they could no longer do this, the community reconstructed them as failures. The community’s ridicule brought on shame for the participants. This shame did not operate at an individual level alone but extended to kinship networks. This reveals African relational ethics by which identity and personhood are shared across familial ties.

*“He did not receive support; instead, he was laughed at. They would not laugh in his face. They would make sure I heard while I was passing by. It would break my heart to hear how they were talking about my brother.”* (Brother of P6).

Even though it was the recipient alone who misspent his windfall, the community ridiculed his whole family, as others recounted:

*“He sometimes cries to me. Bro, it is bad. People say a lot of things. They even laugh at my family. They say you will not believe that someone from that family blew through money. That is what most of them say. Now that we are in this situation, they say my family deserves everything we went through. It really breaks my heart.”* (Brother of P9).

*“We became laughing stocks. To a point that people mocked us and said we blew our money and we were hobos.”* (P10).

These cases illustrate that the communal gaze functions as a form of social regulation of an individual: Praise grants and maintains personhood, and ridicule lets the recipient know that the community has withdrawn their recognition of that individual’s (or their entire family’s) belonging in the community at large. What these narratives reveal is a sequence of social processes through which wealth is converted into moral expectation, and subsequently into grounds for exclusion. First, the community evaluated the recipient’s worth based on their ability and willingness to give. Second, the community continuously monitored behavior through what can be described as a “communal gaze” by using praise to reinforce desirable actions and ridicule to sanction perceived failures. Third, when resources were depleted and the recipient could no longer give, the community withdrew the recognition they had previously granted. This withdrawal was often sudden, as the recipient moved from being admired to being mocked or avoided as soon as it was evident that they had stopped giving. This sequence demonstrates that personhood was not a stable status but one that required constant maintenance through material generosity.

### Theme 4: experience of exploitation and emotional strain

The final theme captures a different side to belonging: one that operated as conditional and was sustained only through continued economic support. The participants found that the bonds that granted them personhood also made them vulnerable to exploitation. The expectations placed on the recipients were open-ended and not easily refusable. Members of the community made excessive demands of the participants to the point of emotional and financial depletion. Generosity became an expectation, rather than a voluntary act.

*“That was another mistake because even if people have money, once you arrive, they do not want to spend. He also had his township friends that he had to buy food for. On the weekends, he had to buy alcohol. Even the soccer players. When he was around, they never wanted to spend their money.”* (Friend of P7).

These demands of maintaining generosity in exchange for the preservation of their social standing inflicted strain on the recipients and a sense of being used rather than being valued. One participant described the abandonment explicitly:

*“That is why, when you lose yourself along the way, they start to call you names. They forget what you have done for them. They are actors in award-winning soapies. I helped them. I gave them a roof over their head and bought them food, but when I was down and out, nobody showed up.”* (P1).

The quote above illustrates a shift from meaningful belonging to a burdensome requirement for maintaining personhood. When they no longer had the money, the recipients were left emotionally depleted and socially exposed. The loss of wealth did not relieve them of their obligation but instead heightened their shame and disappointment. This shows that personhood, once granted through generosity and visibility, became fragile when the individual could no longer fulfill community expectations. The emotional strain they felt was a consequence of identity being tied to giving without boundaries. This reveals a more demanding side to community belonging. Generosity, once voluntary, became an expectation that was difficult to refuse without social consequences. This placed recipients in a bind: If they refused to give, they risked being judged as selfish and losing social standing, but giving excessively depleted their resources. When their resources were exhausted, the recipients were abandoned and ridiculed. This suggests that belonging in this context was not affirming but extractive, requiring constant performance through giving without clear boundaries.

## Discussion

This study examined how sudden wealth reshapes identity in community contexts. The findings show that this shift was enabled by changes in recognition and belonging. Identity was first elevated, then regulated, then strained, and ultimately collapsed. Currently, African communitarianism frames relational personhood as affirming and humanizing, and being recognized by others is central to being seen as a person. However, the data call for a refinement of this framing so that sudden wealth operates as an identity disruption. Mama (2001) criticizes identity discourse that does not account for power and politics. Our findings extend both African communitarian scholarship (Mama, 2001) and sociological work on recognition by demonstrating that recognition may become organized around resource distribution. Participants were valued and included when they could provide material benefits to others. Once those resources diminished, recognition was withdrawn despite their previous social standing. In this context, personhood remained dependent on continued access to economic resources. Personhood must be theorized as something conditional, revocable, and extractive. It is currently seen as something that can be earned through moral participation, but it has not been tested against contexts of sudden wealth. Our data shows that personhood is granted conditionally, and if one’s giving meets the satisfaction of those around them, then the individual is allowed to retain their personhood. However, when their resources are depleted, their personhood is then withdrawn, regardless of the moral standing they may have previously enjoyed. Therefore, relational personhood disciplines behavior through praise and ridicule. We learn through the data that, although personhood may be granted through moral action, it requires constant maintenance through giving without boundaries. This challenges what we understand of African communitarianism and aligns closely with critical scholarship that acknowledges power and vulnerability within the community.

The findings also call for a shift in the conceptualization of sudden wealth. Scholarship currently views it as a financial windfall that has psychological effects on the recipient. However, the recipients of the financial windfall exemplify how sudden wealth can be perceived as an identity shock that reorganizes social hierarchies and moral expectations. In this way, sudden wealth is a catalyst for accelerated identity shifts and makes cracks in the socially conferred identity visible. Existing sudden wealth scholarship seldom accounts for how fast and reversible these identity transitions may be in unequal, collectivist contexts. This study has filled this gap.

Lastly, within identity scholarship, there needs to be greater consideration of how belonging depends on continued access to and distribution of resources. Currently, it is regarded as affective and symbolic in nature (Çolak et al., 2025; Imboden, 2024). The findings of the present study suggest that belonging is closely tied to recognition, and recognition is tied to access to resources. The individuals in *I Blew It* were valued because they could provide financial support and opportunities to others. Once this capacity diminished, their inclusion in the wider community became fragile. Belonging therefore operated as a socially negotiated outcome linked to resource distribution. Thus, individuals are treated as sites through which resources are expected to flow to those who desire them, and if they oblige, they can be seen as important and valuable to the community. This value comes from their ability to give money, ease financial burdens where applicable, and pay for goods for others. Once their money is depleted, their value diminishes along with it. In this context, belonging is instrumental and shapes how identity is maintained in the community. Put differently, social inclusion ceases to be granted by virtue of living in the community but instead depends on what the recipient can provide to community members in the moment. So, while identity is performed through speech and status claims, it is also enacted through financial acts under the gaze of the community. This complicates idealized views of relational identity by showing its fragile nature. It is important to highlight that the above implications hold for the South African context, which is both a deeply unequal society and collectivist in nature. Therefore, depending on their own contexts, not all communities will regulate personhood and identity in similar ways.

### Extending theories of cultural processes and recognition

Our findings contribute to the cultural sociology of inequality in three ways. First, while Lamont et al. (2014) theorize that inequality is produced through everyday cultural processes such as identification and evaluation, their focus is largely on how these processes operate in routine, taken-for-granted ways. We show that in contexts of sudden wealth, these processes operate with unusual speed and visibility. Identification shifts rapidly from celebration to stigmatization as resources decline, and evaluation is directly tied to the recipient’s capacity to provide material support. This suggests that in highly unequal, collectivist settings, cultural processes may be intensified because material resources are scarce and those who have them become socially visible.

Second, our study extends the research of Kahana and Schmid (2026) on recognition and negation claims. Kahana and Schmid examine how elites defend their moral worth in the face of criticism by making recognition claims (“I am good”) and negation claims (“You are wrong to criticize me”). Our participants, however, were not elites defending a stable position. They were ordinary people who experienced a temporary elevation in status and then lost it. We found that recipients in this context rarely made successful recognition or negation claims. Instead, they experienced a withdrawal of recognition from the community, which they were largely unable to reverse. This suggests that the capacity to make effective moral claims is itself stratified. Those with sustained resources and social power can craft counter-narratives; those whose wealth is temporary and who remain embedded in unequal, resource-scarce communities cannot.

Third, our findings challenge idealized accounts of relational personhood in African communitarianism. While this tradition emphasizes that personhood is earned through moral participation in community life, our participant accounts show that personhood can also be conditional, extractive, and punitive. In this context, community recognition does not simply affirm identity; it disciplines it. Individuals are praised for giving and shamed for not giving. This aligns with critical perspectives (Mama, 2001) that caution against romanticizing community as a unified, supportive entity. We therefore argue that theories of recognition must attend to how scarcity and inequality shape the conditions under which belonging is granted or withdrawn.

### Why this case matters beyond South Africa

Our findings are grounded in the South African context, marked by high inequality and strong collectivist values. However, the mechanisms we identify are not unique to South Africa. Conditional granting of personhood based on resource distribution, the use of communal gaze to regulate behavior, and the withdrawal of recognition when resources decline are likely to emerge in any context where material scarcity and strong communal obligations coexist.

What makes the South African case theoretically useful is that it makes these mechanisms visible. In contexts where inequality is less extreme or where individualism is stronger, the withdrawal of recognition may be more subtle or take different forms. However, the underlying logic—that belonging depends on continued resource distribution—may still operate. By studying an extreme case, we can see processes that might otherwise remain hidden. This is the basis of our claim that our findings offer theoretical insights that carry across contexts.

### Practical implications

The findings have implications for sudden wealth interventions. When individuals gain windfalls, they might be expected to practice self-protection, enforce boundaries, and refuse to provide financial support, which are all reasonable, socially acceptable behaviors that should be mastered to maintain financial stability. However, the findings of this study challenge this notion, given the expectations that were placed on the recipients. Therefore, support for people who attain sudden wealth needs to include a reframing of refusal as an ethical rather than a selfish act. In this way, recipients should be able to normalize refusal as a moral duty to themselves and their goals while retaining their ability to continue to give back to the community in responsible, sustainable ways. For example, recipients should be empowered to teach those who seek unreasonable support that preserving resources protects long-term communal stability, that funds are finite, and that giving unsustainably harms their dependents. Although the preservation of funds and self should not be unfairly seen as anti-community, our data shows that it can be interpreted as a withdrawal from communal responsibility and therefore be interpreted as a threat to personhood. For this reason, support structures must help recipients reframe refusal as stewardship over funds, as opposed to a moral failure.

Setting and enforcing the previously mentioned boundaries comes at a social cost that recipients need to be aware of. They may experience rejection and accusations of selfishness and of lacking Ubuntu, a communal belief that we are through others and that regards our humanity as being shaped by how we interact with others as codependent beings (Letseka, 2012; McCluskey and Lephalala, 2010). Recipients might internalize this backlash as their personal failures, rather than expected responses to what is perceived to be a breaking of shared values or expectations. Recipients will require protection of their sense of wealth and social standing by anticipating and naming this expected backlash and preparing emotionally for the status decline that will accompany it. Failure to do so will result in shame and loss of self once they can no longer be providers.

Interventions must also acknowledge that power asymmetries exist within the community, which has the power to grant and withdraw personhood. This study demonstrates that community is not only supportive, as is normally framed in literature, but also demanding and extractive, as is often seen in migration scholarship (e.g., Alowais and Suliman, 2025; Shamase and Sekaja, 2025). It can also be punitive when communal expectations are not met, a phenomenon rarely studied in research about community. It is therefore important to acknowledge that care and coercion coexist and that, equally, recipients require protection that enables them to preserve their personhood while continuing to participate in communal life. Practically, recipients can do this through investing in people and assets in the community and formalized support agreements. This way, expectations are contained, and the recipients’ personhood is protected, as their generosity and commitment to the community remains visible.

### Limitations and directions for future research

This study has a few limitations. Ideally, identity transitions should be studied as they unfold over time. Future research should follow recipients through a longitudinal study. This would allow researchers to understand better how personhood is negotiated months or years after wealth decline. Only through such a research design may scholars begin to see whether diminished recognitions stabilize or recover, or whether social standing is permanently altered.

Our focus on a single Southern African context prioritized theoretical advancement over cross-cultural comparison. Although we gained an in-depth understanding of the phenomenon, future research should examine whether similar patterns of conditional personhood emerge in other unequal, collectivist societies across the Global South.

It is also important to acknowledge that the narratives analyzed in this study were mediated through television production processes. *I Blew It* is a reality documentary series edited and designed to emphasize dramatic trajectories, particularly stories that involve windfalls and subsequent losses. Production choices, including which stories are foregrounded and how participant accounts are edited, impact the narratives that viewers, including us as researchers, ultimately see. Because of this, the experiences portrayed may be dramatized for effect. Furthermore, participants are aware of being filmed. They may adjust their self-presentation and manage their impressions accordingly, performing for the camera or emphasizing certain aspects of their stories to align with the show’s storytelling structure (see McGowan and Sekaja, 2022). While we cannot access behind-the-scenes production decisions, we have treated the documentary narratives as accounts that reflect both the participants’ experiences and the editorial choices, rather than as transparent records of experience. This means that we analyzed what the participants said about their identities and work, rather than treating their accounts as direct evidence of behavior. Future research could complement these mediated narratives with in-depth interviews to explore how individuals narrate their experiences and make sense of their identities outside of a televised setting.

A further limitation concerns the composition of the sample. All 10 cases included in the analysis involved male recipients of sudden wealth. This reflects the broader composition of the television series in which the vast majority of recipients featured were men. The findings therefore primarily show how recognition, belonging, and community expectations were experienced by men who gained and later lost wealth. Future research should examine whether women who experience sudden wealth encounter similar expectations and obligations regarding social standing.

## Conclusion

This study examined how sudden wealth changes whom people are seen to be in their communities in South Africa. We found that personhood is granted on the condition that people continue to share their resources, and it is withdrawn when they stop sharing. This leads to shame and a broken sense of self. By bringing together identity theory and African communitarianism, we demonstrated that while belonging to a community can be affirming, it can also be demanding and conditional. These findings help to explain how inequality shapes recognition and belonging in the collectivist society of South Africa.

## Data Availability

Publicly available datasets were analyzed in this study. The data analyzed in this study are available on Showmax, a subscription-based streaming platform. The authors accessed the episodes through personal Showmax subscriptions. The paywalled nature of the source does not affect replicability, as other researchers may similarly subscribe to access the same content. Further inquiries can be directed to the corresponding author.
